# Migration and Transformation of Multiple Heavy Metals in the Soil–Plant System of E-Waste Dismantling Site

**DOI:** 10.3390/microorganisms10040725

**Published:** 2022-03-28

**Authors:** Jianming Lu, Ming Yuan, Lanfang Hu, Huaiying Yao

**Affiliations:** 1 Key Laboratory of Green Chemical Engineering Process of Ministry of Education, School of Environmental Ecology and Biological Engineering, Wuhan Institute of Technology, Wuhan 430073, China; 21916010030@stu.wit.edu.cn; 2 Zhejiang Key Laboratory of Urban Environmental Processes and Pollution Control, Ningbo Urban Environment Observation and Research Station, Chinese Academy of Sciences, Ningbo 315800, China; lfhu@iue.ac.cn; 3 Key Laboratory of Urban Environment and Health, Institute of Urban Environment, Chinese Academy of Sciences, Xiamen 361021, China

**Keywords:** e-waste, heavy metal, PLFA, ^13^C-PLFAs, microbial community structure

## Abstract

E-waste generation has become a major environmental issue worldwide. Heavy metals (HMs) in e-waste can be released during inappropriate recycling processes. While their pollution characteristics have been studied, the migration and transformation of different multi-metal fractions in soil–plant system of e-waste dismantling sites is still unclear. In this study, pot experiments were conducted to investigate the migration and transformation of different multi-metal fractions (Cu, Pb, Zn and Al) in the soil–plant system using two Chinese cabbage cultivars (heavy metals low-accumulated variety of Z1 and non-low-accumulated Z2) treated with or without biochar. The result showed that the acid-soluble fraction of Cu, Pb, Zn and Al in soil decreased by 5.5%, 55.7%, 7.8% and 21.3%, but the residual fraction (ResF) of them increased by 48.5%, 1.8%, 30.9% and 43.1%, respectively, when treated with biochar and plants, compared to that of the blank soil (CK). In addition, Pb mainly existed as a reducible fraction, whereas Cu existed as an oxidisable fraction. Biochar combined with plants significantly increased the ResF of multi-metals, which reduced the migration ability of Pb among all other metals. The relative amount of labelled ^13^C in the soil of Z1 was higher than that of Z2 (25.4 fold); among them, the Gram-negative bacteria (18-1ω9c, 18-1ω7c) and fungi (18-2ω6c) were significantly labelled in the Z1-treated soil, and have high correlation with HM migration and transformation. In addition, *Gemmatimonadete* were significantly positive in the acid-soluble fraction of HMs, whereas *Ascomycota* mostly contributed to the immobilisation of HMs. Therefore, the distribution of fractions rather than the heavy metal type plays an important role in the HM migration in the soil–plant system of e-waste dismantling sites.

## 1. Introduction

With rapid developments in the field of electronics and information technology, the disposal of electronic waste (e-waste) has been regarded as a serious environmental problem [1]. At present, China is one of the largest producers and consumers of e-waste, with 7.2 Mt produced during 2017, causing a dilemma both in terms of quantity and toxicity of its components [2]. The incineration residues, broken particles, wastewater and dust generated during the recovery of e-waste will enter the local ecosystem through atmospheric subsidence and rainwater runoff [3,4]. Heavy metals (HMs) present in e-waste can be released during inappropriate recycling processes [5]. Most recycling centres are widely distributed in remote areas, which poses considerable risk to farmland security and environmental health [6,7]. Heavy metal(loid)s are non-biodegradable and can only be transferred from one chemical state to another. Furthermore, they are highly persistent in the soil, and can be accumulated in plants and animals [8,9,10]. The speciation of HMs in soil is typically divided into four fractions according to the European Community Bureau of Reference (BCR): Acid-soluble fraction (AF), reducible fraction (RF), oxidisable fraction (OF) and residual fraction (ResF) [11]. Among all the fractions, the AF of HMs was adept at transformation and diffusion, which may release them into the ecosystem [12,13]. In addition, the change in soil pH and redox potential (Eh), and the transformation of different HM speciation would render the non-residual fractions bioavailable [14,15]. Despite the fact that many studies have focused on the heavy metal migration in tailing soil (the movement of heavy metals in soil), the migration and transformation of different fractions of multiple heavy metals in e-waste dismantling sites, especially in the soil–plant system, has received little attention.

The sorption/desorption dynamics of HMs in soil are principally influenced by soil pH, Eh and the soil mineral content such as metal hydroxides, phosphates, clays, metal oxides and organic/inorganic matter [16,17,18,19]. These soil physicochemical properties are easily affected by different agricultural management practices, such as the biochar amendments [20]. Biochar is a carbon-rich substance that is derived from organic feedstock and produced through limited oxygen thermal combustion [21]. It has been widely applied because of its high cation exchange capacity, rich carbon content, stable structure and large surface area [22]. Recently, biochar has been potentially used as a soil amendment for improving soil physical–chemical characteristics and decreasing the HM transformation. For instance, the addition of biochar derived from sewage sludge/cotton stalks decreased the bioavailable forms of Cu in the soil by 34.9% [23]. In addition, biochar efficiently reduced high levels of soluble Zn and Cd in contaminated soil [24]. However, considering the various fractions of HMs, the role of biochar on the migration and transformation of different fractions of multi-metals is inadequately understood, especially at e-waste dismantling sites.

The migration of different HM speciation in rhizosphere soil is not only related to the soil physical–chemical properties, but may also be influenced by soil microbial community. Microbes are cosmopolitan in nature and may live in various habitats, including those in harsh environmental conditions [25]; many can form biofilms that help withstand harsh environmental conditions, such as aridity and high temperatures, through extracellular and intracellular sequestration, permeability barrier exclusion, cellular target sensitivity reduction, enzymatic detoxification, efflux pumps and other mechanisms [26]. The abundance of soil bacteria and fungi is significantly correlated with HMs [27,28]. For instance, it was found the AF of Pb was the main fraction affecting bacterial community structure, whereas the OF and RF of Zn and Pb were dominant factors influencing fungal community in a typical Pb-Zn mining site in Hanyuan, Sichuan, China [29]. Recently, various methods have been developed for studying the microbial community composition in soil; the majority of them are unknown and are yet to be cultivated [30]. In particular, different bacterial groups exhibit varied patterns of phospholipid fatty acids (PLFAs), which allows for direct microbial community identification [31]. PLFA analysis can also evaluate the metabolically active proportion of the microbial population when combined with stable isotope probing (SIP) after incubating with ^13^C-labelled substrates [32,33,34]. In addition, the use of high-throughput pyrosequencing allows for a more detailed research on soil microbial communities and a better understanding of their taxonomic diversity [35,36,37]. The combined use of ^13^C-labelled PLFA and high-throughput pyrosequencing may provide further information about the soil microbial community structure. However, little is known about the relationship between the various HM fractions and the microbial community composition, richness and evenness, especially in e-waste dismantling sites.

Previous studies have indicated that disassembling e-waste resulted in substantial heavy metal pollution in nearby farms, causing potential health hazards to residents [38,39]. Vegetables reportedly contributed ≥70% of Cd uptake in human bodies [40]. Chinese cabbage (*Brassica chinensis* L.) is one of the most extensively cultivated green vegetables in China, the arable land of which reached 2.67 million hectares, with a yield of 1.38 billion tonnes in 2005 [41,42,43]. In addition, Chinese cabbage is considerably specific in the uptake of heavy metal compared with that of other crops [44]. However, little attention has been directed toward multi-metal migration in Chinese cabbage around an e-waste dismantling site. Therefore, this study aims to (1) explore the migration and transformation of multiple heavy metal fractions in rhizosphere soil of e-waste dismantling sites using Chinese cabbage treated with and without biochar, (2) understand the correlation between microbial communities and the different HM fractions in e-waste dismantling sites. This study makes a novel contribution to the field of e-waste remediation by being the first to investigate the migration and transformation of fractions of multiple heavy metals (Cu, Pb, Zn and Al) in soil–plant systems by employing two Chinese cabbage cultivars (low accumulated Z1 and non-low-accumulated Z2), and understanding relationships between microbial communities and the different HM fractions using continuous ^13^CO_2_ labelling combined with high-throughput sequencing.

## 2. Materials and Methods

### 2.1. Study Area and Sample Preparation

The soil was collected from Wenling District, Taizhou City, Zhejiang Province, which is one of the typical e-waste processing centres. Local average annual rainfall was 1480–1530 mm, with a humid-rainy climate [45,46]. The specific sampling site was located beside a quarry (121°21′36.396′′ N, 28°32′12.408′′ E), with an elevation of 12 m. The collected soil was air-dried in a laboratory for two weeks, ground and passed through a 20-mesh sieve. Rice straw biochar was obtained by pyrolysing rice straw in a tube furnace at 500 °C for 40 min and passing it through a 2-mm sieve. The basic physical–chemical properties of soil and biochar are shown in Table 1. While Al is not categorised as a heavy metal, it is nonetheless considered in this study because of its high soil background value.

### 2.2. Experimental Design

The experiment was designed with six treatments and three replications, as follows: (1) Blank soil (CK); (2) rice straw biochar (RB) 2.5%; (3) Chinese cabbage of New Beijing 3 as low accumulated cultivar (Z1) [43,47]; (4) Chinese cabbage of Beijingxiaoza 56 as non-low accumulated cultivar (Z2); (5) rice straw biochar and New Beijing 3 (RB-Z1); (6) rice straw biochar and Beijingxiaoza 56 (RB-Z2). The treatments are shown in Table 2. Air-dried soil (400 g) was placed in plastic pots (8.4 cm diameter, 8 cm height). The experimental soil was uniformly treated with biochar (RB) at 2.5% rate on a dry soil basis. Seeds of Chinese cabbage with similar germinating status were selected and sown in plastic pots. After 4 days, the germinated plants were thinned to accommodate five plants in each experimental unit. During the 28-day pot experiment, the Chinese cabbage grew in a regulated and consistent environment: The temperature was maintained at 25 °C with 12/12 day/night regime, and the soil water content was maintained by supplying water at approximately 70% of field capacity.

At a total CO_2_ concentration of 400 ppm, steady-state labelling of photosynthate using ^13^CO_2_ (at approximately 2.0 atom percent excess) began after 12 days of development. The flow rates of CO_2_-free air, ambient CO_2_ (Xinhang Gas Company, Fuzhou, China) and ^13^CO_2_ (Sigma–Aldrich, St. Louis, MO, USA) into the plant development chamber were 18.0 L min^−1^, 7.28 mL min^−1^ and 0.072 mL min^−1^, respectively, to reach the desired CO_2_ concentration. The ambient air (JUN-AIR, Wheeling, IL, USA) was compressed with a compressor and CO_2_ was eliminated using an FT-IR purge gas generator (Parker Hannifin Corporation, Cleveland, OH, USA) to obtain CO_2_-free air.

### 2.3. Analysis of Soil Physical–Chemical Properties

Soil pH was measured in a 1:5 (*w*/*v*) aqueous solution. The total N, C were determined using Flash 2000HT Elemental Analyzer from 50.0 mg freeze-dried soil. Freeze-dried soil (3 g) was entirely mixed with 15 mL of 0.5 M K_2_SO_4_ by shaking for 1 h and filtered using a quantitative filter. Dissolved organic carbon (DOC) and dissolved organic nitrogen (DON) were detected using a total organic carbon analyser (Multi-N/C 2100S; Analytik Jena, Jena, Germany). After dichromate oxidation, soil organic matter (SOM) was measured using FeSO_4_ titrimetry [48]. After being digested with sodium hydroxide, the total K in soil was analysed by flame photometry. In addition, ammonium acetate was used to extract available K. After being digested with HNO_3_-HClO_4_-HF (*v*/*v*/*v*, 4:1:1) at 240 ± 10 °C, the concentrations of heavy metals in soil samples were measured using an inductively coupled plasma optical emission spectrometer (ICP-OES, Optima 8000, PerkinElmer Inc., Waltham, MA, USA) [49]. The four fractions of HMs were obtained according to the optimised BCR sequential extraction method with a slight modification [50], and the detailed steps of the method are shown in Table 3. Among them, the acid-extractable fraction (AF) easily migrates and it has the highest toxicity, while the residual fraction (ResF) is stable and has the weakest toxicity. The reducible fraction (RF) and the oxidisable fraction (OF) are potentially bioavailable to some extent [12,13].

### 2.4. Plant Analysis

After the pot experiment, the plants were collected and the roots were soaked with 0.01 M EDTA for 30 min to remove HMs adhering to root surface. Deionised water was used to wash the plant samples three times and the fresh weight of roots, stems and leaves of plants were measured, respectively. Then, the plant samples were oven dried for 2 h at 105 °C and then dried at 75 °C to get a constant weight. After being digested with HNO_3_-HClO_4_ (4:1), the concentration of multiple metals in plants was determined by ICP-AES (6300; Thermo Fisher, Waltham, MA, USA).

### 2.5. Phospholipid Fatty Acid (PLFA) Analysis

Lipid extraction and PLFA analysis were carried out using the modified Bligh and Dyer method [51,52]. Briefly, 1.0 g freeze-dried soil was eluted continuously and nitrogen dried by adding with Bligh–Dyer soil extract (chloroform-methanol) under the protection of citric acid buffer. Then, the phospholipids were separated from other lipids on the LC-Si SPE tube. Finally, the phospholipid fatty acid methyl ester was extracted with a mixture of n-hexane and chloroform. A gas chromatograph with a flame ionization detector (GC-FID, Agilent Technologies, Santa Clara County, CA, USA) and a MIDI Sherlock Microbial Identification System were used to identify and quantify the FAMEs (MIDI Inc., Newark, DE, USA) [53]. As detailed by Thornton [54], the δ^13^C of individual PLFAs was evaluated using a Trace GC Ultra gas chromatograph with combustion column coupled through a GC Combustion III to a Delta V Advantage isotope ratio mass spectrometer (Thermo Finnigan, San Jose, CA, Germany).

### 2.6. DNA Extraction and Illumina Miseq Sequencing

FastDNA^®^ SPIN Kit (MP Biomedicals, Santa Ana, CA, USA) was used to extract soil DNA from 0.5 g of well-mixed samples according to the manufacturer’s procedure. The V3–V4 region of the 16 s rDNA gene was amplified using the universal primers 515F (5′-GTGCCAGCMGCCGCGG-3′) and 907R (5′-CCGTCAATTCMTTTRAGTTT-3′). The ITS gene was amplified using the universal primers ITS1F (5′-CTTGGTCATTTAGAGGAAGTAA-3′) and ITS2R (5′-GCTGCGTTCTTCATCGGATGC-3′). After a 5 min denaturation step at 94 °C, 30 cycles of denaturation at 94 °C for 30 s, annealing at 52 °C for 30 s, elongation at 72 °C for 30 s and a final extension step at 72 °C for 10 min were performed. PCR amplicon libraries were prepared for Miseq sequencing. In brief, PCR reactions contained 2 μL genomic DNA (10 ng/μL), 25 μL of 2 × GoTaq Green master mix (Promega, Madison, WI, USA), 1 μL of 10 mM of forward and reverse primers containing barcodes and 21 μL of milli-Q water. PCR conditions included a 5 min initial denaturation at 94 °C, 30 cycles of 30 s denaturation at 94 °C, 30 s annealing for bacterial 16S rDNA at 52 °C and fungal ITS region at 60 °C, 30 s elongation at 72 °C and a 10 min final extension step at 72 °C. Following the manufacturer’s instructions, PCR products were purified using a TIANgel Midi Purification Kit (Tiangen Biotech, Beijing, China) and quantified using a NanoDropTM 2000 spectrophotometer (Thermo Scientific, NY, USA). Purified products were pooled together for library preparation using a NEBNext^®^UltraTM DNA Library Prep Kit (New England Biolabs, MA, USA). An Illumina MiSeq PE250 platform (Majorbio Bio-Pharm Technology Co., Ltd., Shanghai, China) was used for the bacterial 16S rDNA and fungal ITS sequencing. Quality control and software stitching were performed on the raw data obtained from high-throughput sequencing, and the adapter primers and barcode were removed with cut adapt in QIIME2. DADA2 in QIIME2 was applied to denoise and generate the amplicon sequence variants (ASVs) using QIIME2 according to the methods described by Gao et al. [55]. Taxonomy was assigned using the sequences available in the SILVA database (138) for 16S rRNA gene sequencing data and UNITE database (12.11) for fungal ITS region sequencing data.

### 2.7. Statistical Analyses

All data were analysed and displayed based on average values using Origin 2018. Significant differences were analysed using SPSS 22.0 (SPSS Inc., Chicago, IL, USA) software with one-way ANOVA and Wallen–Duncan. The differences were considered to be statistically significant for *p* < 0.05. Redundant analysis (RDA) on abiotic and biotic variables was performed using CANOCO (V4.5, Biometris, Wageningen, The Netherlands) based on data of the Bray–Curtis distance for microbial communities and Euclidean distance for soil physical–chemical properties and HM concentrations.

## 3. Results

### 3.1. Soil Physicochemical Properties, Heavy Metals and Al Concentration in Soil and Plant under Different Treatments

pH values, SOM, total nitrogen (TN) and DOC of rhizosphere soil treated with biochar or plants are summarised in Table 4. The results show that the soil chemical properties were altered by biochar and plants (Z1, Z2). Furthermore, with biochar treatment, the TN content in soil decreased by 5.9%, while soil DOC increased by 3.4% compared with that of the CK. In addition, the DOC content in rhizosphere soil of Z1 significantly increased from 513.78 mg·kg^−1^ to 571.58 mg·kg^−1^, while that of Z2 was reduced by 13.6% compared with that of CK.

**Figure 1:**
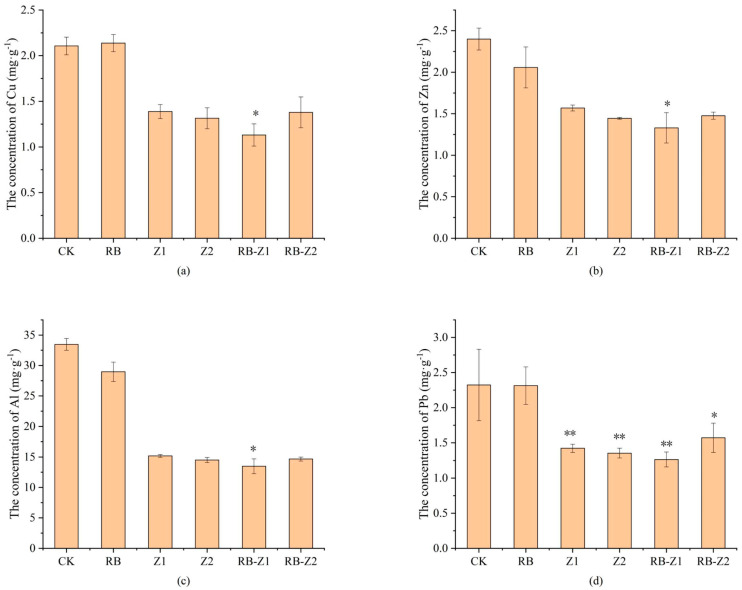
Concentrations of (**a**) Cu, (**b**) Zn, (**c**) Al and (**d**) Pb in rhizosphere soils under different treatments. The data are means ± SD, *n* = 3. Asterisks (* and **) denote a value significantly greater than the corresponding control value (one asterisk means *p* < 0.05 and two asterisks indicate *p* ≤ 0.01).

The HM and Al concentrations in soil with different treatments are shown in Figure 1. Compared to the CK, Zn and Al concentrations in soil decreased 14.3% and 13.4% in biochar-amended soil. In addition, when treated with plants and biochar (Z1, Z2, RB-Z1 and RB-Z2), Pb, Cu, Zn and Al concentrations in soil significantly decreased by 88.8%, 46.3%, 44.6%, and 59.7%, respectively, compared with CK (Figure 1, *p* < 0.05). Among all treatments, the lowest heavy metal concentration in soil was found with RB-Z1, especially for Cu, Zn, Al.

**Figure 2:**
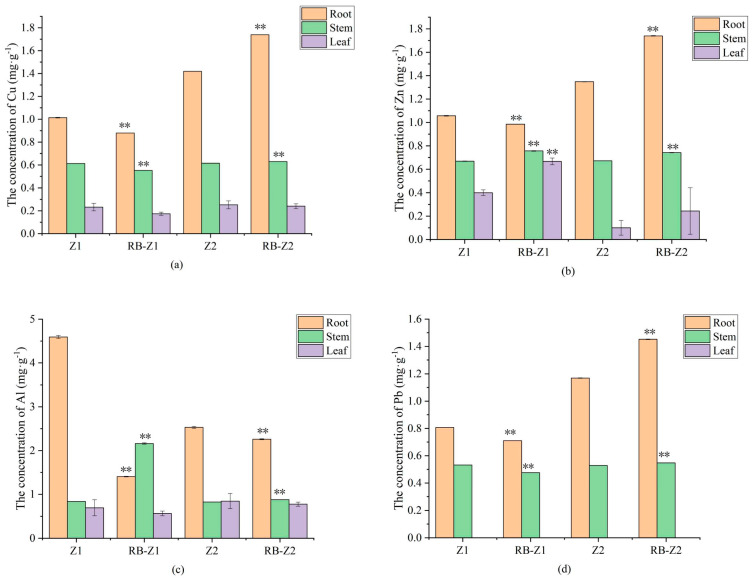
Concentrations of HMs and Al in roots, stems and leaves of plants grown in e-waste dismantling soils amended with biochar or not. (**a**) Cu, (**b**) Zn, (**c**) Al and (**d**) Pb. The data are means ± SD, *n* = 3. Asterisks (**) denote a value significantly greater than the corresponding control value (*p* *≤* 0.01).

Compared with the data obtained in the control group with the treatment, biochar amendments significantly increased the fresh weight of Z1 by 64.8% (*p* < 0.05), and reduced heavy metal concentrations in its root (12.0%, 6.8%, 13.2% and 69.4% for Pb, Zn, Cu and Al, respectively) (Figure S1 and Figure 2). In the case of stem, compared with Z1, Cu and Pb concentrations significantly decreased, by 9.8% and 10.5% under the treatment of RB-Z1, respectively. In addition, Cu and Al concentrations in the leaf of RB-Z1 reduced by 25.0% and 18.3%, respectively, when compared with Z1. Combining the data, Z1 displayed lower metal accumulation and transportation ability than that of Z2.

### 3.2. Effect of Biochar and Plants on the Metal Fraction Distribution in Soil of E-Waste Dismantling Sites

**Figure 3:**
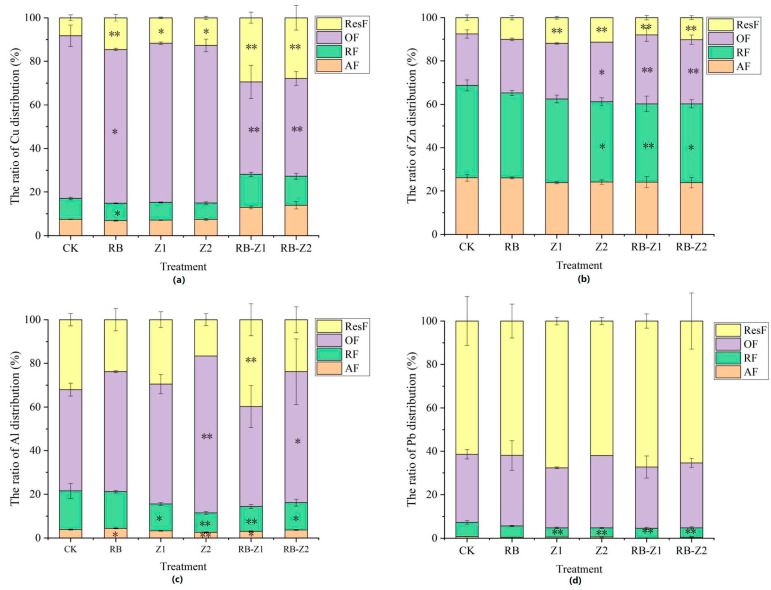
Distribution and transformation of multiple metals in rhizosphere soil under different treatments, (**a**) Cu, (**b**) Zn, (**c**) Al and (**d**) Pb. The data are means ± SD, *n* = 3. Asterisks (* and **) denote a value significantly greater than the corresponding control value (one asterisk means *p* < 0.05 and two asterisks indicate *p* ≤ 0.01).

Biochar treatment and plants considerably altered the fraction distributions of the HMs in the rhizosphere soil (Figure 3). Compared to the CK, the ratio of ResF of Cu and Zn with biochar increased by 61.8% and 32.7%, respectively, whereas the ResF of Al reduced by 31.3%. In addition, in the biochar-treated soil, the AF of Pb decreased by 40.0% while the OF of Al increased by 9.7%. Compared to the CK, during the treatment of Z1 and Z2, the ratio of ResF of Cu, Pb and Zn increased by 46.7%, 34.1% and 58.7%, respectively. The OF of Pb and Zn in the rhizosphere soil of Z2 increased by 20.1% and 12.0%. However, the AF of Pb and Zn presented a reduction of 10.0% and 10.6%, respectively. In addition, compared to the CK, the OF of Al in the rhizosphere soil of Z2 significantly increased, reaching 55.0%. Under the treatment of biochar and plants (RB-Z1 and RB-Z2), compared to the CK, the ratio of ResF of Cu, Pb, Zn and Al increased by 48.5%, 1.8%, 30.9% and 43.1%. Considering the four heavy metals together, Pb mainly exists as ResF (reach 67.7%), but Cu usually exists as OF (reach 74.7%).

### 3.3. Microbial Community Structure and Diversity in Rhizosphere Soil under Different Treatments

**Figure 4:**
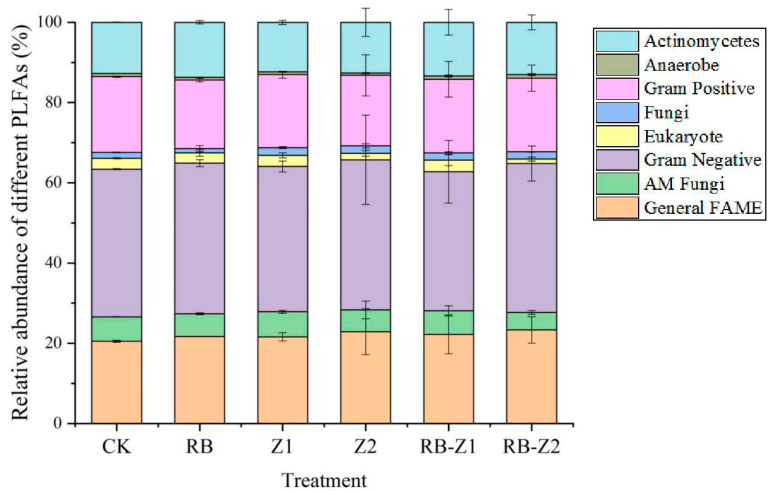
Relative abundance of different phospholipids fatty acids in the rhizosphere soil. The data are means ± SD, *n* = 3.

**Figure 5:**
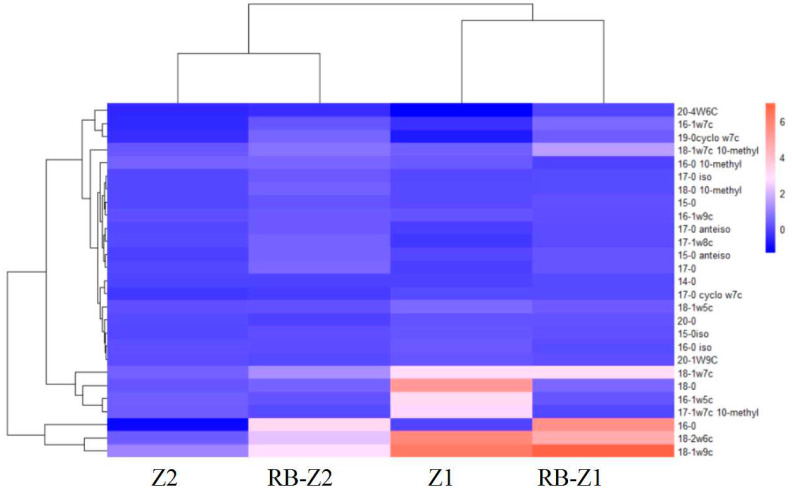
The ^13^C isotope-labeled PLFAs in soil of different treatments.

The relative abundance of different PLFAs in rhizosphere soil is shown in Figure 4. Compared to the CK, Eukaryote and Actinomycetes percentages in RB-Z2 significantly decreased, by 58.8% and 33.4%, respectively, while the relative abundance of Gram-negative bacteria decreased by 5.8%. The results of PLFA-^13^C isotopes in soil are shown in Figure 5. The relative abundance of labelled ^13^C in the soil of Z1 was higher than that in Z2 (25.4 fold). Among them, the Gram-negative bacteria (18-1ω9c, 18-1ω7c) and fungi (18-2ω6c) were considerably labelled under the treatment of Z1, which indicated that these bacteria and fungi prefered to use the root exudates produced by Z1 as carbon source. In addition, general fatty acid methyl ester (FAME) (16-0) could only be labelled in the presence of biochar. Biochar addition reduced the relative abundance of general FAME ^13^C (18-0), AM Fungi ^13^C (16-1 ω5c) and Actinomycetes ^13^C (17-1 ω7c 10-methyl). These microbial communities preferably used soil organic matter as carbon sources. Combined with the results of PLFAs, the relative amount of general FAME (16-0) did not increase with biochar. In the rhizosphere soil, 18-1ω9c, 18-1ω7c, 18-2ω6c and 16-0 were the main labelled PLFAs, and the sum of these four ^13^C-PLFAs accounted for more than 59% of the total labelled PLFA-C.

**Figure 6:**
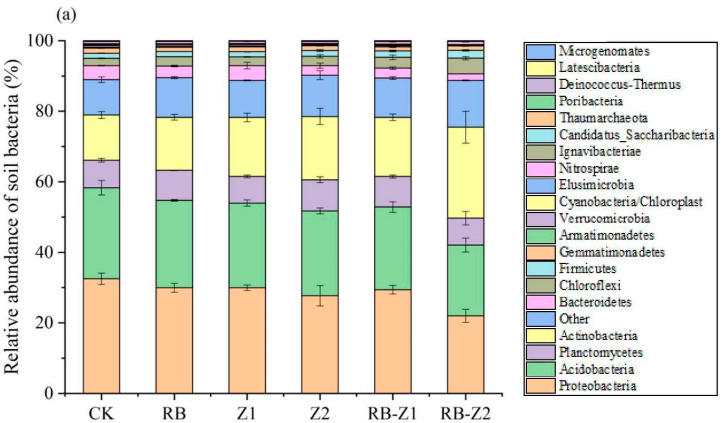

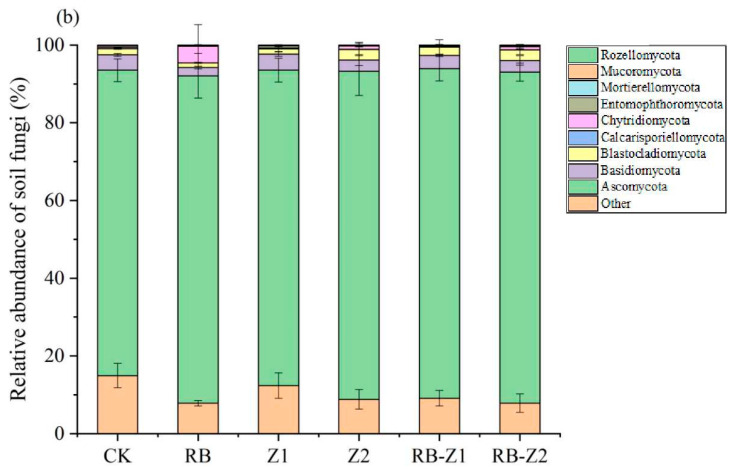
Community composition of (**a**) bacteria and (**b**) fungi at phylum lever in each treatment. The data are means ± SD, *n* = 3.

In total, 1,293,792 16s rDNA and 1,709,263 ITS sequences were obtained (Figure 6), respectively. The average length of the base sequence was 376.62 bp and 241.84 bp. According to the results, the main bacterial phylum was *Proteobacteria* (21.9–31.3%), followed by *Acidobacteria* (20.0–26.4%), *Actinobacteria* (12.4–25.7%) and *Planctomycetes* (7.58–8.77%). When treated with biochar (RB) or plants (Z1 and Z2), the relative abundance of *Proteobacteria* significantly reduced from 31.3% to 27.5%, while *Actinomycoteria* significantly increased from 12.4% to 17.8%. In addition, compared to CK, *Bacteroidetes* significantly reduced from 4.1% to 1.8%, but *Chloroflexi* increased significantly when treated with RB-Z1 and RB-Z2. Considering fungi, *Ascomycota* was the main microbial community in the rhizosphere soil (81.2–85.2%), followed by *Basidiomycota* (2.16–4.12%) and *Blastocladiomycota* (1.19–2.73%). Compared to CK, the relative abundance of *Basidiomycota* decreased by 44.0% and 25.4% in RB and Z2, respectively. In contrast, the relative abundance of *Blastocladiomycota* increased 79.9% and 81.0% in Z2 and RB-Z2, respectively. From all the read sequences, 203 bacterial and 168 fungal genera were identified, and the most common 20 genera are listed in Table S1. The genera majorly varied in their abundance levels in different groups; the genera *Zavarzinella*, *Gaiella*, *Blastopirellula*, *Dothideomycetes*, *Ascomycota* and *Pleosporales* were the most abundant in RB, whereas the genera *Gaiella*, *Dothideomycetes* and *Curvularia* were the most abundant in RB-Z1.

### 3.4. Relationships between the Metal Fractions and the Environmental Conditions

**Figure 7:**
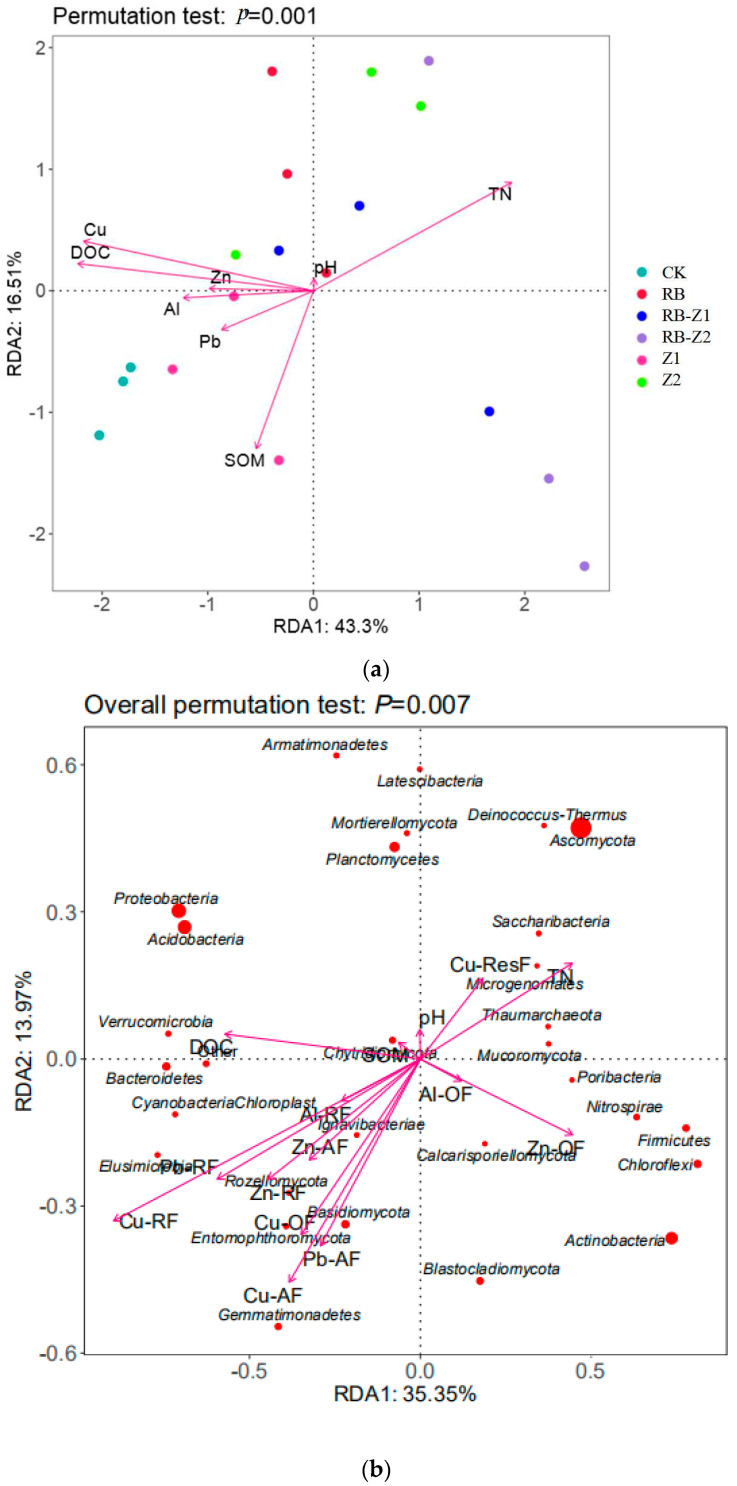
Redundancy analysis (RDA) of samples based on physicochemical characteristics. (**a**) correlation analysis between groups and physicochemical characteristics under different treatments. (**b**) correlation analysis between bacterial and fungal communities and physicochemical characteristics.

The relationships between microbial communities and environmental conditions were determined using RDA and based on the phylum-level information from all rhizosphere soil samples, as shown in Figure 7a. RDA1 and RDA2 contributed to 43.3% and 16.51% of the entire variation, respectively. The RB+Z treatments were majorly located in the right portion of the RDA ordination diagram, which significantly positively correlated with the TN content in the soil. In contrast, the CK, Z1 and Z2 were located in the left region of the RDA ordination diagram.

The correlation between the bacterial and fungal microbial communities in the soil and environmental factors at the phylum level is shown in Figure 7b. RDA1 and RDA2 contributed to 35.35% and 13.97% of the entire variation, respectively. According to the RDA result, the most critical components regulating bacterial and fungal community architectures were soil DOC, TN, the ResF of Cu and the OF of Zn. Most bacterial communities such as *Thaumarchaeota*, *Armatimonadetes* and *Actinobacteria*, and fungal communities such as *Ascomycota* and *Calcarisporiellomycota* were especially positively correlated with soil TN content, and significantly negatively related with soil heavy metal (Cu, Zn, Pb, Al) content. In contrast, *Acidobacteria*, *Poribacteria*, *Bacteroidetes* and *Rozellomycota* were significantly positively correlated with soil heavy metal content and organic matter content (SOM). Besides, *Ascomycota*, *Acidobavteria* and *Proteobacteria* were three central microbial communities; *Ascomycota* was negatively correlated, while *Acidobacteria* and *Proteobacteria* were positively correlated with heavy metal content. Both the RF and AF of four metals were located in the same quadrant, which positively correlated with *Rozellomycota*, *Basidiomycota* and *Gemmatimonadetes*. However, the ResF of Cu significantly positively correlated with TN, *Ascomycota* and *Thaumarchaeota*, which contradicted the other Cu fractions. Furthermore, the AF of four metals were particularly negatively correlated with *Ascomycota*.

## 4. Discussion

### 4.1. Effect of Biochar and Plants on Soil Physicochemical Properties and Metal Concentrations

SOM and DOC are critical indicators used to measure soil quality. Our results revealed that the addition of biochar could increase SOM and DOC in soil (Table 4). It is reported that biochar could increase SOM and DOC contents in soil directly or through a priming effect [56]. Biochar is a stable carbon source that may retain carbon in the soil for an extended period and can help reduce carbon emissions [22]. In addition, the reducing carbon dioxide release and increasing organic matter contents in soil by biochar could lead to the increase of carbon (C) sequestration in soil [57]. Therefore, biochar addition successfully enhanced the soil organic carbon content. Besides, the increase of DOC in soil may also contribute by plant root litter decomposition [58].

The application of biochar is an emerging solution for heavy metal stabilization in soil [59]. Biochar has been proved to exhibit high HM immobilising ability in farmland, mining areas and urban soils [60,61,62]. In this study, biochar demonstrated a higher immobilisation ability toward Zn than that of other HMs. This may partly be because zinc can preferentially precipitate with anions such as hydroxide, carbonate and phosphate on biochar and form complexes with organic ligands in soil [63,64]. Cation exchange and complexation by organic ligands were suggested to be the main mechanisms for the immobilization of Zn by biochar in the soil [65]. In addition, according to a recent study, biochar’s high electronegativity could contribute to the electrostatic attraction of positively charged ions [66,67], and the cations such as Ca and Mg released by biochar can also exchange metal ions on their surface [68].

### 4.2. Migration and Transformation of Different Multi-Metal Fractions in the Soil–Plant System

Notably, biochar addition evidently increased the ResF percentages of Cu and Zn, while the OF of Cu in the rhizosphere soil significantly decreased (Figure 3). Generally, the OF of HMs can combine with the organic matter obtained mainly from the animal and plant residues [69]. Humus, one of the primary forms of soil organic matter, contributed to the encapsulation and chelation of HMs [70]. Biochar, to a certain extent, could promote the transformation of HMs from oxidisable state to residue state. In addition, biochar reportedly contained abundant carboxyl functional groups, which were the primary binding sites for Cu, and explained the increase in residual Cu percentage [71]. Furthermore, the ResF of Pb in the rhizosphere soil of Z1 was higher than that of Z2, indicating that Z1 exhibited higher Pb immobilisation ability. Considering Zn, compared with that of CK, biochar treatment with plant significantly decreased the RF percentage, but increased the percentage of the ResF of Zn. Compared with Z2, Z1 treatment demonstrated a higher Zn immobilisation ability. The interaction of HMs with root exudates is facilitated by functional groups such as phenolic hydroxyl groups (-OH), carbonyl groups (-C=O), carboxyl groups (-COOH) and ester groups (-COOR) present on the biochar surface [72,73,74,75].

Considering Al, its OF significantly increased during treatment with biochar or plants (Figure 3), which may be because the combination of Al with organic carbon derived from biochar was stable [76]. However, the ratio of ResF of Al only increased when treated with RB-Z1, resulting in a decrease in Al accumulation in Z1 (Figure 2 and Figure 3). In addition to the uptake by plants and the secretion of root exudates, the solubility and exchangeability of Al could also be reduced by biochar [77,78,79,80], which would result in the decrement of Al in soil under the treatment of biochar with plants. Al is one of the most abundant elements in soil, accounting for approximately 8% of the total soil minerals [81]. While Al is not considered a heavy metal, its high concentrations can seriously harm the environment. In acidic soils, aluminium transforms into toxic forms such as Al^3+^ and Al(OH)^2+^, which induces toxicity in soil and even plants [82]. Z1 exhibited a lower ability to accumulate Al with the application of biochar than that of Z2, which may result from an increase in ResF-Al. Furthermore, Al, to a certain extent, continued to display similar properties as other HMs. According to previous studies, HM immobilisation in the soil by biochar was mainly due to chemical adsorption [83]; the vast specific surface area of biochar provided more active sites for the adsorption of HMs. Oxygen-containing functional groups (mainly-OH) reportedly dissociated and acquired a negative charge, which electrostatically interacted with positively charged HM ions [84,85].

The AF of heavy metal presented the strongest migration capacity, with the heavy metal RF and OF being referred to as the non-residual state, resulting predominantly from human activities [86]. However, the ResF of heavy metal is difficult to transfer and alter, owing to its origin from natural minerals [46]. Furthermore, most of Cu exists as OF fraction (Figure 3), which is due to the great affinity of Cu for organic matter, resulting in stable chelated compounds [87,88], and the humic substances in coal slime may exhibit a higher affinity for Cu complexation than that of other metals. In contrast, Pb demonstrated an absolute advantage in the ResF fraction in different treatments, which resulted in its lowest migration and transformation ability among all the metals.

### 4.3. Microbial Role in the Migration and Transportation of Multiple Metals

Various soil environmental parameters influence soil microbial communities [89]. In this study, bacterial and fungal community composition changed in response to biochar–plant treatments and varied soil physicochemical properties. *Proteobacteria*, *Acidobacteria*, *Planctomycetes* and *Actinobacteria* were the major phylum performing essential roles in the bacteria community in all six treatments (Figure 6 and Table S1). These bacteria are more common in low-nutrient soils, and have been discovered in several HM-contaminated soil settings [90,91,92,93]. The treatment of biochar or plants was correlated with the increase of the relative abundance of *Actinobacteria*. *Proteobacteria* was reportedly correlated with the carbon and nitrogen cycle [94,95]. In this study, *Proteobacteria* were positively correlated with DOC, and negatively correlated with TN and the OF of Zn (Figure 7b). In addition, the relative abundance of *Chloroflexi* increased, while that of *Bacteroxidetes* decreased in RB (Figure 6a). *Bacteroxidetes* was positively correlated with SOM and DOC, while *Chloroflexi* was positively correlated with the OF of Al and Zn. At the genus level, the relative abundance of bacterial taxa in different groups differed significantly (Table S1). The relative abundance of two *Bacillus* (*Blastopirellula* and *Bacillus*, belonging to *Firmicutes*), two *Actinomycetes* (*Gaiella* and *Nocardioides*), *Arthrobacter* and *Flavobacterium* significantly increased during RB+Z treatments when compared with CK (Table S1). This was consistent with the study results of Lan [96], wherein *Acinetobacter* and *Bacillus* were often used for HM detoxification, and would resist the harm caused by HMs via biosorption and extracellular transformation. Specifically, they can produce various viscous colloidal substances for the passivation of HMs [97]. Besides, *Gaiella* were associated with DOM and Cr-resistant chemical diversity in paddy soils [16]. *Arthrobacter* and *Flavobacterium* reportedly resisted high concentrations of HMs and promoted plant growth [98]. In addition, it is reported that some Gram-negative bacteria have a heavy metal homeostasis mechanism and could code for a complex that transports metals from the periplasmic space across the outer membrane [99,100], which may also decrease the harm caused by heavy metals. Therefore, soil physicochemical properties and heavy metal types determine the changes of soil microbial communities, and the dominant bacteria selected by the environment in turn reduce the harm of heavy metals, thereby the soil system could achieve a dynamic balance.

In terms of fungi, the relative abundance of *Chytridiomycota* and *Blastocladiomycota* increased during the application of biochar and Z2, respectively (Figure 6b). At the genus level, compared with that of the control, the relative abundance of many fungal genera in the *Ascomycetes* class, such as *Bipolaris*, *Curvularia*, *Penicillium*, and *Scytalidium* significantly increased when treated with biochar and plants (Table S1). According to previous reports, arbuscular mycorrhizal fungi reveals an inseparable connection with plant roots in HM-contaminated soils [101]. In addition, they also play an essential role in the processes involved in heavy metal tolerance, accumulation and its transfer from plant root to shoot [102]. Compared with arbuscular mycorrhizal fungi, endophytic fungi not only reduced the toxicity of pollutants by degrading, transferring or immobilising them in the soil, but also improved plant tolerance toward heavy metal, thereby promoting plant growth [103]. *Bipolaris* sp was reportedly beneficial in enhancing the resistance of seeds to polymetallic (Al, Cd, Cu, Pb, Zn) stress [104]. In addition, *Penicillium funiculosum* LHL06 reportedly enhanced the resistance to Cu stress in *Glycine max* L, increasing the plant biomass and enriching plant roots [105]. Considering the results, since fungi were easily labelled with the ^13^C-PLFA in Z2, which presented the lowest metal transport ability, they play an important role in reducing the migration and transformation of HMs in the soil–plant system.

The microbial community composition varies in long-term polluted soils [106]; Nevertheless, such changes are dependent on both soil HMs and chemical characteristics [107]. RDA was used to examine the relationships between HM fractions and soil chemical characteristics, with bacterial and fungal communities in this study. The soil bacterial and fungal communities were influenced by different soil chemical properties and HM concentrations. For the soil chemical properties, the microbial community was mainly influenced by soil TN and DOC. *Ascomycota* positively correlated with TN, while *Protebacteria* and *Acidobacteria* positively correlated with DOC; these three fungi and bacteria were the major microorganisms. TN is an essential index that helps evaluate soil fertility, and is also one of the important elements necessary for plant growth [108]. Besides, DOC plays a vital role in regulating the carbon cycles in the soil ecosystem [109], DOC is a mixture of low-molecular-weight humic chemicals with many carboxyl groups, which contribute in creating stable metal complexes [110,111]. It affects the migration and transformation of HMs in the soil through a series of reactions such as ion exchange, complexation, adsorption, chelation, redox and flocculation [112,113]. In addition, as one of the carbon sources, DOC would be rapidly used by certain microorganisms, which would then be released by certain algae to achieve a dynamic balance [114].

From another perspective, the RF of Pb, Cu, Zn and Al, the AF of Pb, Cu and Zn and the OF of Cu are mostly located in the third quadrant, and are positively correlated with *Gemmatimonadetes*, *Basidiomycota*, *Rozellomycota*, *Entomophthoromycota*, *Ignavibacteriae* and *Elusimicrobia*. In contrast, the ResF of Cu is located in the first quadrant, and significantly positively correlated with *Ascomycota*, *Microgenmates*, *Candidatus* and *Saccharibacteria*. Therefore, changes in the bacterial community were mainly driven by the combinations of soil heavy metal fractions and various chemical properties. In this study, *Ascomycota* and *Basidiomycota* were the most abundant phyla as previously reported [8,115]. *Ascomycota* is the largest fungal group [116], and the members of *Ascomycota* and *Basidiomycota* in mangrove ecosystems exhibit important ecological functions, such as plant litter decomposition and energy flow [117,118]. Liu [119] proved that the reducible fraction (RF) evidently plays an important role in scavenging HMs. Furthermore, AF is the most vulnerable to environmental impact, and contributes the most to migration and transformation of multiple HMs [120]. Moreover, while the RF is more stable than AF, its absorption and diffusion by plants is hazardous [121]. Therefore, *Basidiomycota* is beneficial to the migration and transformation of HMs, while *Ascomycota* may contribute to HM immobilisation. While different metals exist as various fractions, the effects of environmental factors to the same fraction of different metals are similar. Therefore, the fraction distribution rather than the type of HMs is an important factor that determines the migration of HMs in the soil–plant system. Among them, the AF and RF of heavy metal can easily migrate; in contrast, the ResF of heavy metal has a relatively low mobility. Therefore, effectively increasing the proportion of the ResF of HM is an important approach in reducing their migration and transportation in the soil–plant system. However, a further study focusing on the response mechanism of rhizosphere microorganisms to the transformation and transport of various metal fractions in the soil–plant system should also be carried out.

## 5. Conclusions

The migration and transformation of multi-metals (Pb, Cu, Zn and Al) in the soil–plant system in e-waste dismantling sites could be decreased by biochar supplementation and plants. Generally, Cu and Al mainly exist as OF, whereas Pb exists majorly as ResF and possesses lower migration ability. Compared with CK, the concentrations of Cu, Pb, Zn and Al in Z1 root decreased in some extent, and the ResF of Cu, Pb and Al noticeably increased. During the different treatments of biochar and plants, the lowest heavy metal migration ability was observed for the combination of RB-Z1. In addition, the results of ^13^C-PLFAs labelling revealed that the Gram-negative bacteria (18-1ω9c, 18-1ω7c) and fungi (18-2ω6c) may be associated with the migration and transformation of HMs. Furthermore, *Acidobacteria* and *Proteobacteria* were significantly positively correlated with the ResF of metals. Effectively increasing the ResF proportion of heavy metal is an important way to reduce their migration and transportation in the soil–plant system in e-waste dismantling sites. However, the response mechanism of rhizosphere microorganisms to the transformation of various metal fractions in soil and plant in a long period of time still needs further study.

## Figures and Tables

**Table 1:** Basic physical and chemical properties of soil and biochar.

	Soil	Biochar
Sand (0.05–2 mm)%	85.4 ± 3.1	/
Silt (0.002–0.05 mm)%	13.9 ± 2.5	/
Clay (<0.002 mm)%	0.660 ± 0.067	/
Available P (mg/kg)	7.29 ± 0.44	/
Available K (mg/kg)	13.8 ± 0.5	1.05 ± 0.131
Total K (mg/kg)	113 ± 7	6.53 ± 0.287
Volume weight (g/cm^3^)	0.690 ± 0.131	/
Al (mg/kg)	13,242 ± 118	13.7 ± 0.8
Cu (mg/kg)	2361 ± 285	0.164 ± 0.005
Zn (mg/kg)	1405 ± 660	2.71 ± 0.15
Pb (mg/kg)	2805 ± 846	0.375 ± 0.019
Ni (mg/kg)	83.4 ± 15.2	n.d.
Mg (mg/kg)	398 ± 26	12.0 ± 0.4
Mn (mg/kg)	1262 ± 286	0.796 ± 0.049
Fe (mg/kg)	14,137 ± 4638	12.4 ± 0.7
C/N	22.2 ± 0.7	50.4 ± 0.7
pH	8.15 ± 0.01	10.5 ± 0.3

**Table 2:** The description of different treatments.

Treatments	CK	RB	Z1	Z2	RB-Z1	RB-Z2
description	Blank soil	2.5% rice straw biochar	Chinese cabbage of New Beijing 3 (low accumulated cultivar)	Chinese cabbage of Beijingxiaoza 56 (non-low-accumulated cultivar)	rice straw biochar and New Beijing 3	rice straw biochar and Beijingxiaoza 56

**Table 3:** Analytical conditions for the modified BCR sequential extraction procedure.

Step	Operational Definition	Extract Reagents and Conditions		
		Reagents	Temperature (°C)	Time (h)
1	Acid-extractable (AF)	Dry sample (0.50 g) + 0.1 M CH_3_COOH_2_ (20.00 mL)	22 ± 5	16
2	Reducible fraction (RF)	AF residue + 0.1M NH_2_OH·HCl (20.00 mL, pH 1.5)	22 ± 5	16
3	Oxidisable fraction (OF)	RF residue + 30% H_2_O_2_ (10 mL, pH 2.0)	85 ± 0.5	1
		A second 30% H_2_O_2_ (10.00 mL, pH 2.0) addition and heated with intermittent agitation, then cool	85 ± 0.5	1
		Followed, add 1 M NH_4_OAc (pH 2.0) to make up the volume to 50.00 mL	22 ± 5	16
4	Residual fraction (ResF)	OF residue + 4:1:1 HNO_3_/HClO_4_/HF (*v*/*v*/*v*) (HF, 1.00 mL)	240 ± 10	10

**Table 4:** Physicochemical properties of soil samples under different treatments.

	CK	RB	Z1	Z2	RB-Z1	RB-Z2
pH	7.15 ± 0.01 a	7.22 ± 0.04 a	7.01 ± 0.03 b	7.10 ± 0.07 ab	7.09 ± 0.09 ab	7.18 ± 0.05 a
SOM (mg·g−1)	241 ± 3 a	249 ± 6 a	247 ± 6 a	227 ± 10 a	235 ± 5 a	236 ± 20 a
TN (%)	0.630 ± 0.008 ab	0.593 ± 0.019 abc	0.640 ± 0.016 a	0.590 ± 0.020 bc	0.575 ± 0.005 c	0.573 ± 0.037 c
DOC (mg·kg−1)	514 ± 19 abc	531 ± 40 ab	572 ± 19 a	444 ± 43 cd	466 ± 15 bcd	435 ± 45 d

The data are means ± SD, *n* = 3. Different letters in the same column represent significant differences at *p* < 0.05.

## Data Availability

Not applicable.

## Supplementary Materials

The following supporting information can be downloaded at: https://www.mdpi.com/article/10.3390/microorganisms10040725/s1, Table S1. Relative abundance of bacteria and fungi at genus level in rhizosphere soil, Figure S1. Fresh biomass of plants in e-waste dismantling soils under different treatments. Values with the same letter are not significantly different within different treatments (*p* < 0.05).

## Abbreviations

HM, heavy metal; ResF, residual fraction; AF, acid-soluble fraction; RF, reducible fraction; OF, oxidisable fraction; PLFA, phospholipid fatty acid; SIP, stable isotope probing; DON, dissolved organic nitrogen; DOC, dissolved organic carbon; RDA, Redundant analysis; TN, total nitrogen; SBD, soil bulk density; SWHC, soil water holding capacity.
