# Supplementary material for: Migration and Transformation of Multiple Heavy Metals in the Soil–Plant System of E-Waste Dismantling Site
Source: Microorganisms. 2022 Mar 28;10(4):725. doi: 10.3390/microorganisms10040725 (PMC9030041; doi:10.3390/microorganisms10040725)
Supplement: Supplementary file 1 [file microorganisms-10-00725-s001.zip › microorganisms-1637841-supplementary.pdf]

Supplementary Materials:

Table S1

Relative abundance of bacteria and fungi at genus level in rhizosphere soil

Genus(bacteria)	CK	RB	Z1	Z2	RB-Z1	RB-Z2
Unassigned	46.994%	47.961%	47.574%	47.071%	46.858%	46.811%
Gp6	13.698%	12.665%	11.535%	11.868%	11.798%	8.942%
Gaiella	4.569%	5.806%	6.574%	6.707%	6.052%	9.812%
Gp3	3.810%	3.036%	3.634%	2.887%	2.783%	2.534%
Gp4	2.730%	2.670%	2.210%	2.330%	2.413%	1.394%
Blastopirellula	1.960%	2.683%	1.987%	2.810%	2.813%	2.991%
Ohtaekwangia	1.340%	1.006%	1.347%	0.630%	0.717%	/
Zavarzinella	1.280%	1.307%	1.197%	1.380%	1.336%	0.925%
WPS-1_genera_incertae_sedis	0.962%	0.668%	0.680%	0.858%	/	0.523%
Reyranella	0.946%	0.802%	1.023%	0.809%	0.905%	0.568%
Blastococcus	0.939%	0.916%	1.204%	1.247%	1.203%	1.877%
Bradyrhizobium	0.929%	0.741%	0.839%	0.936%	0.940%	0.572%
Aridibacter	0.925%	0.978%	0.846%	0.960%	0.986%	0.680%
Hyphomicrobium	0.888%	1.100%	0.934%	1.125%	1.356%	1.165%
Gemmata	0.855%	0.929%	0.936%	0.921%	0.934%	0.746%
Chryseolinea	0.750%	0.761%	0.861%	/	0.736%	/
Gp16	0.750%	1.077%	1.060%	1.227%	1.210%	1.847%
Gp17	0.707%	0.681%	0.715%	0.861%	0.710%	0.551%
Pirellula	0.666%	/	/	0.657%	/	/
Nitrospira	0.606%	0.627%	/	/	0.750%	0.869%
Nocardioides	/	0.647%	0.812%	0.843%	0.884%	1.744%
Solirubrobacter	/	/	0.737%	0.793%	0.718%	1.161%

Genus(fungi)	CK	RB	Z1	Z2	RB-Z1	RB-Z2
Ascomycota-Stachybotryaceae-Other	12.591%	8.981%	11.746%	8.212%	15.761%	5.906%
Fungi-Other	11.690%	7.826%	12.364%	8.837%	9.113%	7.849%
A-Sor-Hyp-Other	9.651%	7.597%	8.929%	7.812%	7.889%	8.143%
A-Sor-Hyp-Nectriaceae-Fusarium	7.111%	5.971%	6.392%	5.576%	4.820%	5.859%
A-Sor-Hyp-Hypocreaceae-Trichoderma	7.079%	3.846%	6.515%	3.846%	4.576%	3.594%
A-Sor-Other	6.914%	5.649%	6.914%	18.184%	5.848%	7.272%
A-Doth-Other	4.337%	8.598%	4.341%	6.781%	6.809%	6.436%
A-Sor-Hyp-Nectriaceae-Other	3.199%	4.741%	3.028%	4.893%	3.772%	6.755%
A-Doth-Ple-Ple-B-Curvularia	3.087%	5.398%	4.412%	5.600%	6.163%	4.899%
A-Sor-Con-Con-Coniochaeta	3.020%	2.602%	2.938%	4.226%	4.990%	6.435%
A-Sor-Hyp-B-Nectriaceae-nd	2.092%	/	1.688%	1.116%	/	1.695%
A-Other	1.989%	2.027%	3.200%	1.314%	2.164%	3.483%
A-Doth-Ple-Other	1.877%	1.504%	1.424%	1.103%	1.243%	1.141%
A-Sordariomycetes;nd;nd;nd	1.604%	1.490%	/	/	0.886%	/
Bla-Bl-Bl-Cat-Catenaria	1.510%	/	1.173%	2.620%	2.103%	2.308%

A-Sor-Sor-B-Chae-Other	1.340%	5.032%	1.140%	2.114%	4.066%	4.233%
A-Eur-Eur-Asp-B-Aspergillus	1.237%	1.237%	1.055%	0.879%	0.864%	/
Bas-Aga-Can-Cantharellales_fam_Incertae_sedis;Burgoa	1.160%	/	1.486%	/	/	/
A-Sor-Mic-Mic-Pseudallescheria	1.120%	/	1.074%	0.878%	/	1.248%
A-Pez-Pez-Pyronemataceae;nd	0.968%	/	/	0.801%	0.980%	2.563%
Chy-nd;nd;nd;nd	/	3.926%	/	/	/	/
A-Sor-Sor-Other	/	2.970%	/	/	0.970%	/
A-nd;nd;nd;nd	/	2.215%	1.428%	/	1.236%	/
A-Leo-Hel-Hel-Scytalidium	/	1.162%	1.104%	0.950%	1.489%	2.006%
A-Doth-Ple-Ple-B-Bipolaris	/	/	/	0.822%	/	0.973%
A-Sor-Sor-Sor-B-Other	/	/	/	/	/	1.881%

CK, blank soil; RB, rice straw biochar; Z1, low accumulated cultivar (New Beijing 3); Z2, non-low accumulated cultivar (Beijingxiaoza 56); RB+Z1, biochar and New Beijing 3; RB+Z2, biochar and Beijingxiaoza 56.

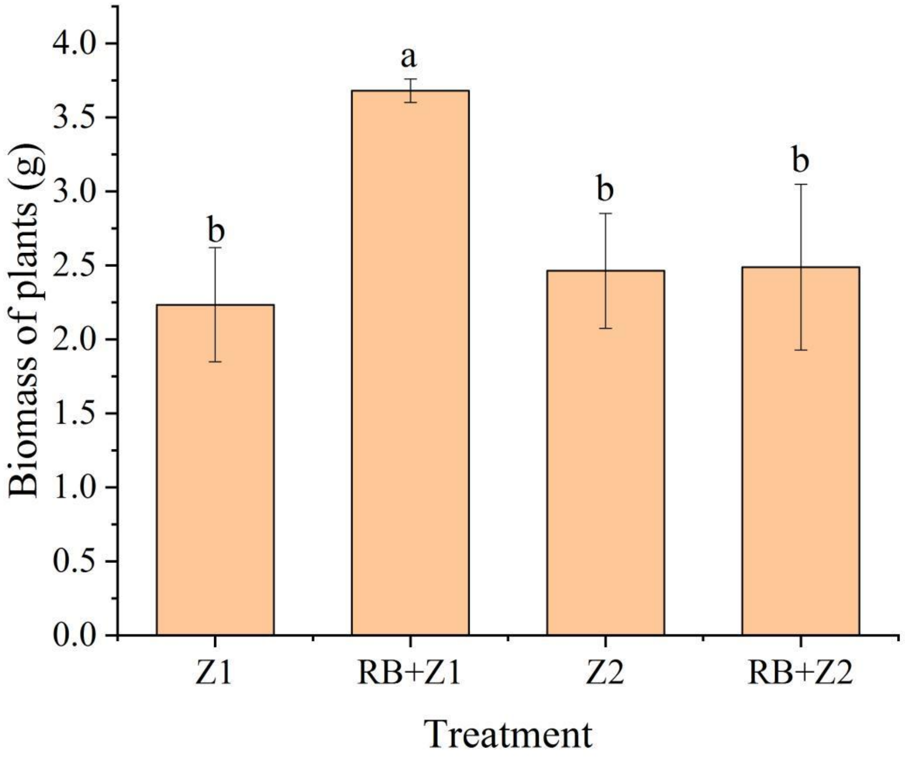

Figure S1. Fresh biomass of plants in e-waste dismantling soils under different treatments. Values with the same letter are not significantly different within different treatments ($p < 0.05$)
